# Preparation of halloysite nanotube-supported gold nanocomposite for solvent-free oxidation of benzyl alcohol

**DOI:** 10.1186/1556-276X-9-282

**Published:** 2014-06-02

**Authors:** Xiaobo Fu, Zhan Ding, Xuan Zhang, Wanliang Weng, Yongjun Xu, Junxu Liao, Zhenkui Xie

**Affiliations:** 1 Department of Energy and Chemical Engineering, Dongguan University of Technology, 1st, University Road, Songshan Lake District, Dongguan 523808, China; 2 Dongguan Key Laboratory of Distributed Energy System, Dongguan University of Technology, 1st, University Road, Songshan Lake District, Dongguan 523808, China

**Keywords:** Nanocomposites, Microstructure, Oxidation

## Abstract

Gold nanoparticles supported on halloysite nanotubes (Au/HNTs) were prepared by a homogeneous deposition-precipitation method. The specific characteristics of the catalyst were characterized in detail, in relation to their performance for solvent-free oxidation of benzyl alcohol. The particular structure of the catalyst resulted in high catalytic activity and stability compared with other supported gold catalysts. The enhanced catalytic activity of the Au/HNTs catalyst was mainly attributed to the presence of a higher amount of oxidized gold species and the tubular structure of the HNTs.

## Background

Selective oxidation of alcohols to more valuable aldehydes, ketones, and carboxylic acids is of great importance to both the fine chemical industry and academia [1]. Numerous stoichiometric oxidizing reagents have been involved to accomplish this transformation, such as dichromate and permanganate. However, these reagents have many drawbacks, such as being toxic, expensive, and un-recyclable. Thus, the developments of a heterogeneous solid catalyst that can use molecular oxygen as a primary oxidant have attracted much more attention. In this context, a series of noble metal supported catalysts for aerobic oxidation of alcohols have been exploited over the last decades.

Among the noble metal supported catalysts, gold supported catalysts have been paid more and more attention, owing to their unique catalytic properties under mild conditions, such as CO oxidation, hydrocarbon combustion, selective oxidation, and water gas shift reaction [2-5]. It is generally accepted that the catalytic performance of the gold catalysts strongly depended on not only the size of the gold particles but also the nature of the support material, the preparation method, and the activation procedure during the synthetic process [6]. As supports, metal oxides have been employed, giving outstanding performance because of their facile activation of molecular oxygen [2,7,8]. At the same time, liquid-phase alcohol oxidation requires addition of soluble bases (metal carbonates, acetates, or borates), especially when inert supports such as silica, carbon, or polymers are used to disperse gold [9].

Halloysite nanotubes (HNTs) (Al_2_Si_2_O_5_(OH)_4_ · 2H_2_O), hydrated layered aluminosilicates of the kaolinite group, containing octahedral gibbsite Al(OH)_3_ and tetrahedral SiO_4_ sheets (i.e., halloysite nanotubes), possess a hollow cylinder formed by multiply rolled layers [10]. Because of their structural features, they offer a potential application as support for catalytic composites and the additive for reinforcing polymers with remarkable, improved mechanical properties and dispersibility. Recently, Yang et al. reported Pd nanoparticles deposited on HNTs nanocomposite for hydrogenation of styrene with enhanced catalytic activity [11]. They cast a new light on using HNTs as catalyst support. Herein, we reported the synthesis of Au/HNTs catalyst and the structure of the catalyst was characterized. The as-synthesized Au/HNTs catalyst showed high catalytic activity for solvent-free oxidation of benzyl alcohol.

## Methods

In a typical procedure, 3.6 g urea was dissolved in 200 mL of 1.46 mmol L^−1^ HAuCl_4_ solution at room temperature. An amount of 0.55 g of HNTs support was then added to this clear solution (5% Au loading), and the temperature of the resulting slurry was increased gradually to 90°C. The temperature was maintained for 4 h, followed by filtering and washing several times with deionized water. The solid product was dried overnight before calcination at 300°C for 4 h in static air.

The crystalline phases were determined using a RIGAKU D/max-2550VB1 18-kW X-ray powder diffractometer (XRD; Shibuya-ku, Japan) with Cu Kα radiation (*λ* = 1.5418 Å). Transmission electron microscopy (TEM) images were obtained using a JEOL JEM-2010 F instrument (Akishima-shi, Japan) equipped with an energy-dispersive X-ray spectroscopy (EDS) at an accelerating voltage of 200 kV. X-ray photoelectron spectroscopy (XPS) measurement was performed using PHI 5600 (Physical Electronics, Chanhassen, MN, USA) with a monochromated Al Kα radiation (*hν* = 1,486.6 eV), calibrated internally by the carbon deposit C 1 s (285.0 eV).

A reactor (50-mL round-bottle flask) was charged with 200 mg of catalyst and 100 mmol of benzyl alcohol. Molecular oxygen was bubbled through the reaction mixture (flow rate = 20 mL min^−1^). The resulting mixture was then heated at 383 K for 8 h and cooled to room temperature. The reaction products were analyzed by a Shimadzu QP5050 GC-MS (Kyoto, Japan).

## Results and discussion

**Figure 1:**
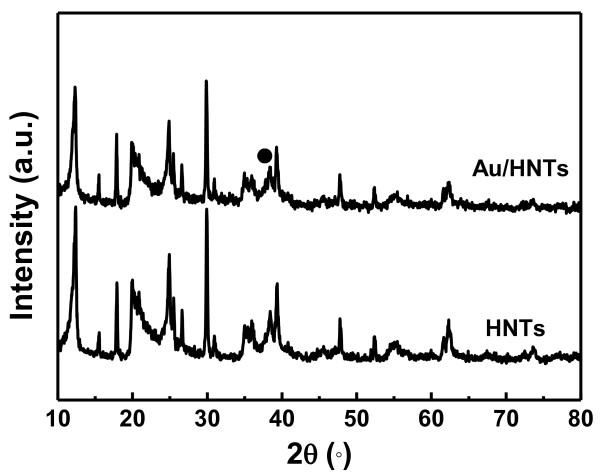
**XRD patterns of HNTs and Au/HNTs.** Black circle, metallic Au.

**Figure 2:**
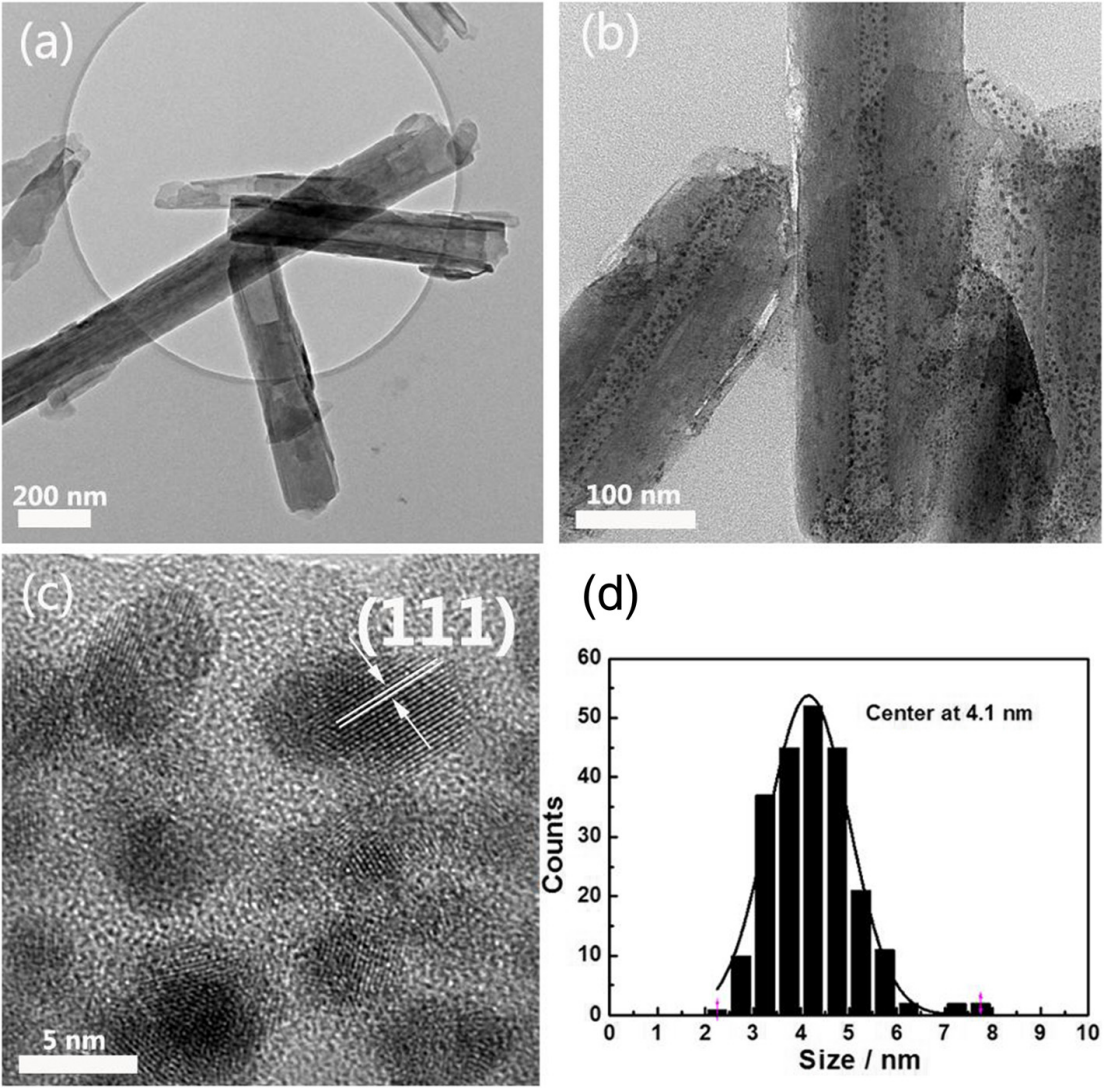
**TEM images of the HNTs and Au/HNTs and size distribution. (a)** Pure HNTs. **(b)** Gold nanoparticles in the HNTs. **(c)** High-resolution TEM image of gold nanoparticles. **(d)** Size distribution of supported gold nanoparticles.

For the HNTs sample, all of the observed peaks are close to the characteristic data of halloysite (JCPDS card no. 29-1487), as shown in Figure 1. For the Au/HNTs sample, all of the observed peaks are almost consistent with those of the pure HNTs, indicating that the whole process of the preparation does not damage the structure of the HNTs. Moreover, considering the overlapping of the diffraction peaks between HNTs and Au particles and the small size of the Au nanoparticles, the metallic gold peaks cannot be well evidenced. Furthermore, due to the tubular structure of the HNTs, the Au nanoparticles mostly filled in the inner tube may also affect the detection of the XRD.To overcome the limitation of the XRD technique, the TEM images of the HNTs and Au/HNTs catalyst are shown in Figure 2. As shown in Figure 2a, white HNTs are short cylindrical hollow tubes averaging 1 to 10 μm in length, with an external diameter of 75 to 150 nm and an internal diameter of 10 to 40 nm. As shown in Figure 2b, a narrow size of gold nanoparticles filled the inner surface of the HNTs or was deposited on the surface of the HNTs. No separate aggregate of the gold nanoparticles was observed in the product, indicating that the nucleation is successfully limited in the inner surface of the HNTs. The high-resolution TEM image (Figure 2c) shows that the distinct crystal structure of the gold nanoparticles was detected, indicating that the gold particles are crystalline. This is in agreement with XRD analysis results. The crystal structure of HNTs remains unchanged after supporting the gold nanoparticles, indicating that the deposition process of gold did not lower the crystallinity of the HNTs. The size distribution of supported gold nanoparticles was evaluated by a statistical measurement of 300 randomly selected particles, which can be found in Figure 2d. These particles are in the range 2 to 8 nm and the average size centers at 4.1 nm.

**Figure 3:**
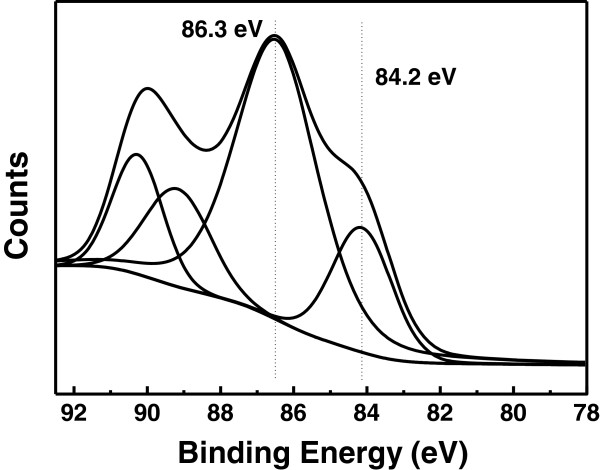
Representative Au 4f core level XPS spectrum of Au/HNTs.

Figure 3 shows the representative Au 4f core level XPS spectrum of the Au/HNTs catalyst. Broad peaks of Au 4f_7/2_ and Au 4f_5/2_ states were observed in the Au/HNTs sample, indicating the presence of both metallic and ionic gold species [12,13]. In addition to the main peak characteristic of metallic Au^0^, the XPS spectra also contain the 4f_7/2_ signals from Au^1+^ ions [12,13]. The deconvolution analysis results of the Au 4f spectra of the Au/HNTs catalysts showed that about 60% of the gold species are oxidized Au^1+^ species. Similar to our findings, Abad et al. have recently shown by XPS and IR spectroscopy the presence of positive gold ions in Au/CeO_2_ catalyst [14]. Such species has been suggested to be of vital importance in the rate-controlling step during the oxidation of alcohols involving the hydride shift from alcohol to gold [15].

**Figure 4:**
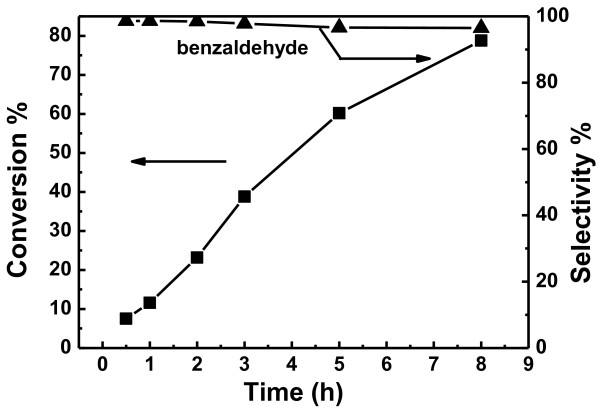
The catalytic performance of the Au/HNTs catalyst as a function of reaction time.

For the Au/HNTs catalyst, solvent-free aerobic oxidation of benzyl alcohol which is often employed as a model reaction for alcohol oxidation was chosen to test its catalytic activity [16-18]. The control experiments using the pure HNTs reveal that less than 2% of the benzyl alcohol can be selectively converted to benzaldehyde within 8 h at 110°C. Figure 4 shows a typical set of results for benzyl alcohol conversion over the Au/HNTs catalyst, illustrating the dependence of conversion and selectivity on the reaction time. As the reaction proceeded, the conversion of benzyl alcohol and the selectivity to benzyl benzoate increased, while the selectivity to benzaldehyde decreased. Enache et al. [17] and Abad et al. [14] have recently reported very high turnover frequency (TOF) values in the solvent-free oxidation of benzyl alcohol at about 100°C for Au-Pd/TiO_2_ (TOF = 607 h^−1^) and Au/CeO_2_ (TOF = 150 h^−1^) catalysts, respectively. To compare with other reported catalysts, the catalytic performance of the Au/HNTs catalyst in the solvent-free aerobic oxidation of benzyl alcohol at 110°C under atmospheric pressure was also investigated. The results showed that the Au/HNTs catalyst exhibited a specific rate of 307 h^−1^ under similar reaction conditions. This value compares favorably with the results reported on Au/CeO_2_ catalysts [17], demonstrating that our catalytic system can serve as a promising catalyst for the selective oxidation of alcohols. Moreover, the Au/HNTs catalyst can be recovered and showed high stability for oxidation of benzyl alcohol.

## Conclusions

In conclusion, we have demonstrated that HNTs are an attractive support for gold nanoparticles, which results in an excellent catalytic activity in solvent-free oxidation of benzyl alcohol. The high catalytic activity is found to be related to the tubular structure of the HNTs and the oxidized gold species. This process is promising in the development of a truly heterogeneous catalyst for alcohol oxidation.

## Competing interests

The authors declare that they have no competing interests.

## Authors' contributions

XBF carried out the synthesis of the materials and drafted the manuscript. ZD, XZ, and WLW participated in the characterization of the materials. The whole project was under the direction of YJX. JXL and ZKX participated in the testing of the catalytic activity of the materials. All authors read and approved the final manuscript.
